# Betatrophin is downregulated in pregnant women with a history of RYGB operation and a high risk of postprandial hypoglycaemia

**DOI:** 10.1038/s41598-020-70075-1

**Published:** 2020-08-04

**Authors:** Michael Leutner, Caspar Matzhold, Luise Bellach, Carola Deischinger, Stefan Thurner, Peter Klimek, Alexandra Kautzky-Willer

**Affiliations:** 1 Unit of Gender Medicine, Clinical Division of Endocrinology and Metabolism, Department of Internal Medicine III, Medical University of Vienna, Waehringer Guertel 18-20, 1090 Vienna, Austria; 2 Section for Science of Complex Systems, CeMSIIS, Medical University of Vienna, Spitalgasse 23, 1090 Vienna, Austria; 3 Complexity Science Hub Vienna, Josefstädter Strasse 39, 1080 Vienna, Austria; 4 Santa Fe Institute, 1399 Hyde Park Road, Santa Fe, NM 85701 USA; 5 IIASA, Schlossplatz 1, 2361 Laxenburg, Austria; 6 Gender Institute, 3571 Gars am Kamp, Austria

**Keywords:** Obesity, Gestational diabetes

## Abstract

Betatrophin is a liver and adipose tissue-derived protein which has recently been linked to glucose metabolism. So far, no data exist about the role of betatrophin in pregnant women with a history of Roux-En-Y gastric bypass (RYGB) operation with a high risk of postprandial hypoglycaemia. In this prospective clinical study, an oral glucose tolerance test (OGTT) and an intravenous glucose tolerance test (IVGTT) were performed between the 24th and 28th week of pregnancy and 3–6 months post-partum in a cohort of obese and normal-weight pregnant women, as well as in women with a history of RYGB operation. In the cohort of pregnant women with RYGB and exaggerated risk of postprandial hypoglycaemic events, basal and dynamic betatrophin levels during the OGTT were lower than in the obese or normal-weight pregnant women (basal levels: 13.66 ± 5.88 vs. 19.03 ± 4.15 vs. 15.68 ± 6.48, p = 0.016; OGTT 60′: 13.33 ± 5.40 vs. 17.37 ± 3.16 vs. 15.84 ± 4.99, p = 0.030). During the OGTT, basal and dynamic betatrophin levels at 60′ were positively associated with glucose levels at 60 min (r = 0.55, p = 0.01 and r = 0.45, p = 0.039). This positive association was followed by significant hypoglycaemic events in the RYGB group. It was only in the RYGB group that betatrophin was negatively related to the disposition index (rho = -0.53, p = 0.014). After pregnancy there was a decrease in basal and stimulated betatrophin levels during the OGTT in all three patient groups. In comparison to normal-weight and obese pregnant women, women with a history of RYGB operation and a high risk of postprandial hypoglycaemic events have lower levels of betatrophin. This indicate a mechanistic role in order to decrease the risk of postprandial hypoglycaemia in this specific cohort.

## Introduction

In recent years, a novel protein expressed in liver and adipose tissue has been discovered: betatrophin, also referred to as RIFL^1^, ANGPTL8^2^ or lipasin^3^. Along with modulations of lipid metabolism^1,2,4^, a positive correlation of betatrophin with postprandial glucose levels has been established in humans^5^ as well as a positive correlation with insulin resistance^6^ and HbA1c levels^7^. A connection to diabetes mellitus has also been established^3,8^, attributing a predictive value for new-onset type 2 diabetes mellitus to betatrophin^9^. Thus also during pregnancy and especially in women with gestational diabetes mellitus (GDM), elevated levels of betatrophin have been reported earlier than in healthy controls^10,11^. Additionally, an association of betatrophin levels with RYGB operation has been observed^12^. In their study, Shankar et al. investigated obese patients with a BMI over 35 kg/m^2^ and type 2 diabetes mellitus undergoing RYGB surgery and could show that just 2 weeks after surgery, betatrophin levels had dropped significantly, accompanied by improved levels of parameters of glucose metabolism such as plasma glucose, insulin and C-peptide^12^. Further, bariatric surgery has been shown to have a diabetes remission rate of about 80%^13^. However, the exact pathophysiological mechanisms behind the significant improvement in glucose metabolism and especially the increased risk of postprandial hypoglycaemia in this specific cohort are not entirely known to date. We have recently shown that glucagon-like peptide 1 (GLP-1) is significantly involved in the regulation of postprandial glucose metabolism, and therefore especially in the increased risk of postprandial hypoglycaemia^14^. However, despite numerous research groups investigating the metabolic implications of an RYGB operation^12–17^, data on betatrophin in certain patient subgroups remain scarce. The aim of the present study was to investigate the role of betatrophin on glucose metabolism in the specific cohort of pregnant women with a history of RYGB operation and high risk of postprandial hypoglycaemia.

## Methods

A detailed description of the methods has been published previously^14,18,19^.

### Study design

The study at hand is a prospective clinical study evaluating glucose metabolism in pregnant women with a history of RYGB operation, obese (OB) pregnant women, and normal-weight (NW) pregnant women at the Medical University of Vienna. The study protocol has been approved by the Ethics Committee of the Medical University of Vienna and all experiments have been carried out in accordance with the declaration of Helsinki. Written informed consent was obtained from all study participants.

### Study participants

In this analysis, 25 females with a history of RYGB surgery, 19 OB females with a body mass index (BMI) of ≥ 35 kg/m^2^ before pregnancy, and 19 NW females with a BMI of < 25 kg/m^2^ before pregnancy were included. The exclusion criteria were bypass surgery other than laparoscopic RYGB, infectious diseases, liver disease or renal disease. The median time span between RYGB and entry to the study was 3.3 years (2.3–5.7).

### Tests and procedures

#### Oral glucose tolerance test (OGTT)

Between the 24th and 28th week of gestation and 3–6 months after pregnancy, a 3 h 75 g OGTT was performed in the morning after a fasting period of at least 8 h. Blood was drawn at 0, 30, 60, 90, 120, 150 and 180 min for the measurement of parameters of glucose metabolism and betatrophin. Hypoglycaemia was defined as blood glucose levels under 54 mg/dl.

#### Intravenous glucose tolerance test (IVGTT)

The IVGTT was performed within 1–2 weeks of the OGTT. At time point 0, 0.3 g/kg of glucose were infused. 20 min after the beginning of the test, a dose of 0.05 IU/kg body weight of insulin was infused. Until 60 min after the start of the IVGTT the plasma concentrations of glucose, insulin and C-peptide were assessed.

### Assays

Betatrophin was analyzed in EDTA plasma samples using the Human Betatrophin Elisa kit from BioVendor (Brno, Czech Republic) according to the manufacturer’s instructions.

### Calculations and statistical analysis

For the calculation of the area under the curve (AUC) for betatrophin the trapezoidal method was used. The total body insulin sensitivity (ISI(comp)) and beta cell function (insulinogenic index) were calculated with the data retrieved from the OGTT. Furthermore, a HOMA-IR index to assess hepatic insulin resistance and a Matsuda index for insulin sensitivity were calculated. The disposition index, a product of the insulinogenic index and ISI(comp), was used to evaluate pancreatic beta cell function in insulin resistance. On the basis of the IVGTT, insulin sensitivity indices, a sensitivity index (SI), and a calculated sensitivity index (CSI) were calculated.

In the statistical analysis, means and standard deviations were used for continuous variables, counts and percentages for categorical variables. Distribution was tested for normality using the Shapiro–Wilk test. Non-normally distributed data were logarithmically transformed. An ANOVA (analysis of variance) was carried out to evaluate the differences between the three study groups. Differences in glucose levels in each group during and after pregnancy were analysed using a paired t-test. For linear dependencies, the Pearson correlation coefficient was used. A p-value of < 0.05 was considered statistically significant.

## Results

The detailed baseline characteristics of the study population have been published previously^14,18,19^. In short, pregnant women with a history of RYGB operation are more insulin-sensitive and are characterized by major alterations in glucose metabolism, including a high risk of postprandial hypoglycaemic events, when compared to obese and normal-weight pregnant women.

**Figure 1:**
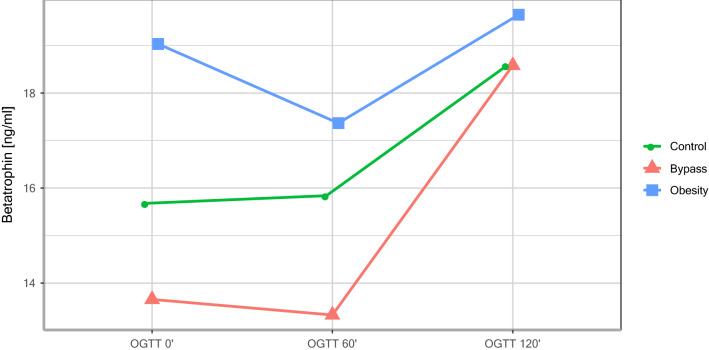
Dynamic changes in betatrophin levels during the OGTT (during pregnancy).

**Figure 2:**
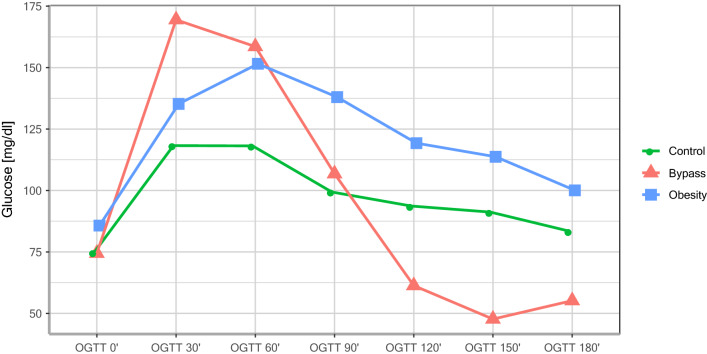
Dynamic changes in glucose levels during the OGTT (during pregnancy).

As presented in Table 1, pregnant women with a history of RYGB operation show significantly lower basal (RYGB: 13.66 ± 5.88 vs. obesity: 19.03 ± 4.15 vs. controls: 15.68 ± 6.48; p = 0.016) and dynamic betatrophin levels during the OGTT (see Table 1 and Fig. 1). In particular, the RYGB group showed significantly lower basal and dynamic betatrophin levels than the obesity group (OGTT 0′: p = 0.016, OGTT 60′: p = 0.030). Figure 2 presents the dynamic changes of glucose values during the OGTT in the three groups, highlighting an increased risk of postprandial hypoglycaemic events in the RYGB group.

**Table 1:** Basal and dynamic betatrophin levels during the OGTT in pregnant women.

Variable	Controls	Bypass	Obesity	p-value^a^
Age (years)	31.11 ± 5.93	31.96 ± 6.97	32.05 ± 4.94	0.867
Betatrophin 0*′*	15.68 ± 6.48	13.66 ± 5.88^b^	19.03 ± 4.15	0.016
Betatrophin 60*′*	15.84 ± 4.99	13.33 ± 5.40^b^	17.37 ± 3.16	0.030
Betatrophin 120*′*	18.58 ± 5.67	18.58 ± 8.94	19.65 ± 4.43	0.858
Betatrophin AUC-OGTT 0*′*–120*′*	1957.67 ± 603.34	1766.86 ± 743.53	2,202.50 ± 417.17	0.095

^a^Group comparison using ANOVA.

^b^Significant vs. obesity.

### Relationship between betatrophin levels and parameters and indices of glucose metabolism (Table 2, 3)

**Table 2:** Relationship between basal betatrophin and parameters and indices of glucose metabolism during pregnancy (significant relationships highlighted in boldface).

Variable	Controls			Bypass			Obesity		
	N	r^a^	p^b^	N	r^a^	p^b^	N	r^a^	p^b^
HbA1c	18	0.31	0.214	13	0.03	0.928	16	− 0.06	0.812
Matsuda Index	19	0.00	0.996	21	− 0.31	0.165	16	− 0.23	0.389
HOMA-IR	19	0.11	0.656	21	0.21	0.356	16	0.17	0.522
Insulinogenic Index	18	− 0.10	0.703	21	− 0.24	0.300	16	− 0.20	0.448
Disposition Index	18	− 0.11	0.656	**21**	**− 0.53**	**0.014**	16	− 0.32	0.230
SI_IVGTT	19	0.21	0.391	20	− 0.11	0.636	17	− 0.24	0.356
DI_IVGTT	19	− 0.01	0.964	20	0.06	0.798	17	− 0.24	0.348
CSI	19	0.25	0.310	20	− 0.10	0.672	17	− 0.26	0.308

^a^Pearson’s correlation coefficient.

^b^p-value against null hypothesis that Pearson’s correlation coefficient is zero.

**Table 3:** Relationship between the AUC of betatrophin (0–120′) during the OGTT and parameters and indices of glucose metabolism during pregnancy (significant relationships highlighted in boldface).

Variable	Controls			Bypass			Obesity		
	N	r^a^	p^b^	N	r^a^	p^b^	N	r^a^	p^b^
HbA1c	17	0.39	0.121	13	0.11	0.721	16	− 0.04	0.881
Matsuda Index	18	− 0.26	0.294	21	− 0.27	0.232	16	− 0.32	0.226
HOMA-IR	18	0.44	0.066	21	0.19	0.400	16	0.20	0.454
Insulinogenic Index	17	0.01	0.965	21	− 0.24	0.303	16	− 0.13	0.635
Disposition Index	17	− 0.31	0.231	**21**	**− 0.49**	**0.024**	16	− 0.26	0.34
SI_IVGTT	18	0.07	0.787	20	− 0.08	0.729	17	− 0.32	0.213
DI_IVGTT	18	− 0.10	0.691	20	0.08	0.743	17	− 0.33	0.190
CSI	18	0.09	0.716	20	− 0.05	0.839	17	− 0.33	0.190

^a^Pearson’s correlation coefficient.

^b^p-value against null hypothesis that Pearson’s correlation coefficient is zero.

**Figure 3:**
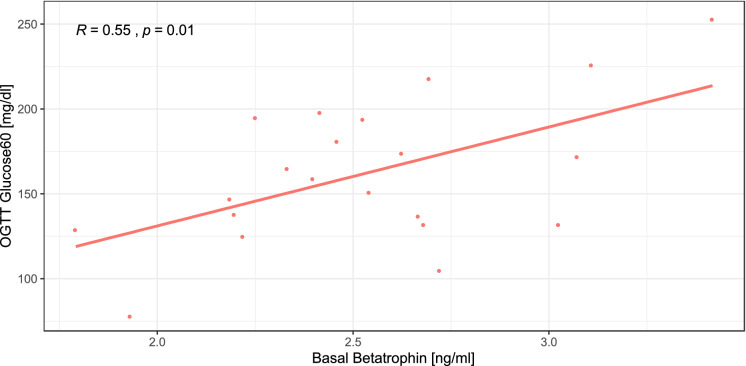
Relationship between basal betatrophin and dynamic glucose levels at 60 min during the OGTT in pregnant women with RYGB.

**Figure 4:**
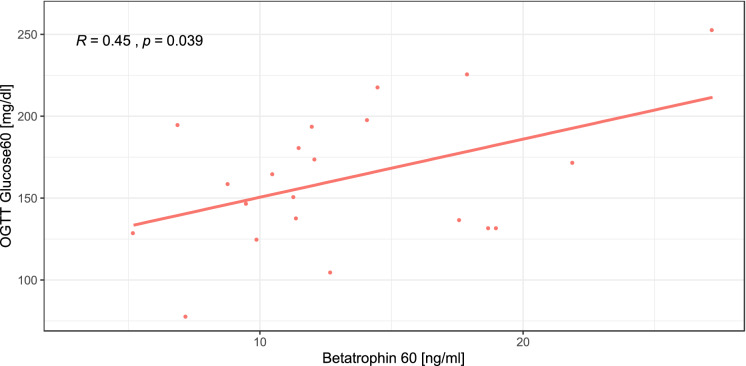
Relationship between dynamic betatrophin and dynamic glucose levels at 60 min during the OGTT in pregnant women with RYGB.

Table 2 shows that a relationship between betatrophin and parameters and indices of glucose metabolism could not be observed either in the control group or in the obese pregnant women. Only in the RYGB group was there a significant negative correlation between basal betatrophin levels and the area under the curve (AUC) during the OGTT with beta cell function in insulin resistance, measured using the disposition index (see Tables 2, 3). Additionally, basal betatrophin and betatrophin levels at 60 min during the OGTT were positively related to glucose levels at 60 min during the OGTT in pregnant women with RYGB (see Fig. 3, 4). This relationship was closely followed by a significant drop in glucose levels at 90 min during the OGTT (see Fig. 2).

### Post-partum

**Figure 5:**
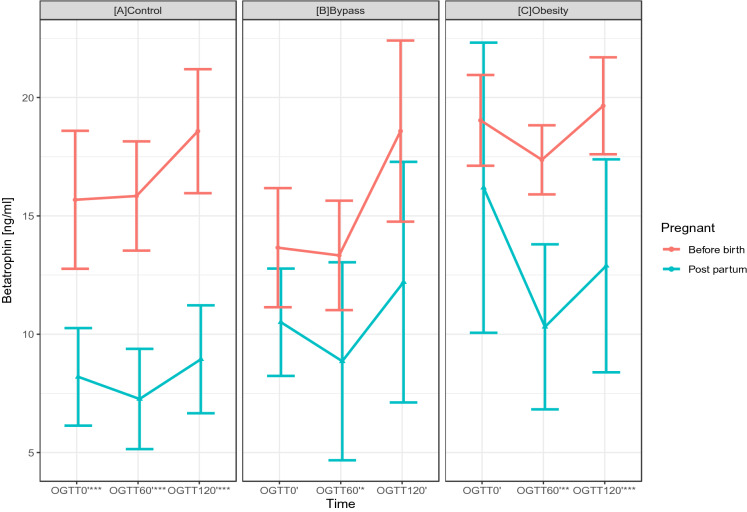
Comparison of betatrophin levels during pregnancy and post partum in the (**a**) control, (**b**) bypass and (**c**) obesity groups (*p < 0.05, **p < 0.01, ***p < 0.001).

As shown in Fig. 5, the dynamic levels of betatrophin decreased significantly in all three study groups after pregnancy, yet the most prominent difference can be observed in the control group, which also showed a significant change in basal betatrophin levels after pregnancy.

## Discussion

To the best of our knowledge, we were able to show for the first time that pregnant women with a history of RYGB surgery have significantly lower basal and dynamic betatrophin levels when compared to obese and normal-weight pregnant women—indicating a possible downregulation to prevent aggravation of postprandial hypoglycaemic events. Interestingly, a positive relationship between betatrophin levels at 60′ with dynamic glucose levels at 60′ during the OGTT could be observed. This positive relationship was closely followed by a significant drop in the glucose values in RYGB patients. Further, we could only find a negative correlation between betatrophin and the disposition index, linking betatrophin to beta cell function in insulin resistance, in the RYGB group. After pregnancy a significant drop in betatrophin levels could be observed in all groups.

In 2012, betatrophin was newly characterized as a triglyceride level-modulating protein mainly expressed in the liver and adipose tissue, also known as ANGPTL8^2^ or lipasin^3,20^. Espes et al. were among the first research groups to detect a positive correlation between betatrophin and diabetes mellitus, arguing that betatrophin secretion is induced by insulin resistance^7,21^. The correlation between betatrophin and diabetes mellitus is backed by several sources^9^^,^ including a positive association with impaired glucose tolerance (IGT)^22^^,^ fasting glucose^23^^,^ insulin resistance^6,24,25^ and 2 h-postprandial plasma glucose levels^5^. Previously, we have shown that pregnant women with a history of RYGB operation are more insulin-sensitive and have an exaggerated risk of postprandial hypoglycaemic events when compared to controls. Therefore, GLP-1 was mainly involved in the regulation of postprandial glucose metabolism, and was the main driver of the increased risk of postprandial hypoglycaemic events in pregnant RYGB patients^14^. However, the exact pathophysiological mechanisms of the increased risk of postprandial hypoglycaemic events are presently not entirely known and earlier studies hypothesized that multiple mechanisms could be related to the dramatic, postoperative changes in glucose metabolism. Interestingly, we could find lower basal and dynamic plasma levels of betatrophin in pregnant women with RYGB when compared to controls in the present study. So in the context of earlier studies which hypothesized that betatrophin could have beta cell proliferating effects, the lower basal levels and the downregulation of betatrophin during the OGTT in the specific cohort of pregnant women with a history of RYGB operation in the present study could be a consequence of negative feedback in order to prevent aggravation of postprandial hypoglycaemic events in this specific cohort, which were present anyway. Interestingly, we could find a positive association between betatrophin and glucose levels at 60 min during the OGTT, indicating that betatrophin is also secreted in a compensatory manner in pregnant RYGB patients, albeit in lower levels when compared to controls. This positive relationship was closely followed by a significant drop in glucose levels, resulting in a dramatic increase in hypoglycaemic events in pregnant RYGB patients. Our results showed that there are quick dynamic changes of betatrophin concentrations during several time-points of the OGTT. However, studies have yet to investigate the exact pathophysiological mechanisms behind the dynamic changes of betatrophin and its release after glucose load and the relationship with the regulation of postprandial glucose metabolism, including increased risk of postprandial hypoglycaemia in pregnant women with a history of RYGB. Hence a possible physiological downregulation of betatrophin in order to prevent a larger drop in glucose levels must be mentioned here. Potential negative feedback between betatrophin and insulin resistance has been discussed earlier in a study by Espes et al., who compared betatrophin concentrations in type 1 diabetic patients and controls^21^. Accordingly, a mouse model and cell culture study ascribed betatrophin a regulatory role in the insulin pathway under prevailing insulin resistance^26^. In the present study, we also found a negative correlation between betatrophin and the disposition index in RYGB patients, which presents the beta cell function in insulin resistance^27,28^. The lower levels of betatrophin in the RYGB group could also indicate a physiological reflex downregulation in order to prevent further increasing the risk of postprandial hypoglycaemic events by improving beta cell proliferation, a mechanism which has been shown in earlier studies. In the present study, obese pregnant women were characterized by having the highest levels of betatrophin. This is an interesting aspect, as it is known that obesity in particular is closely related to impaired insulin sensitivity^29^^,^ an effect also physiologically prevalent in pregnancy^30^. It has long been known that different underlying metabolic states occur during pregnancy, mainly effected by physiologically increased insulin resistance during gestation in order to ensure sufficient glucose and free fatty acid supply to the fetus. This physiologic insulin resistance is induced by increasing levels of the human placental growth hormone (hPGH). Besides, the peroxisome proliferator-activated receptor γ (PPARγ) activity is also suppressed, leading to further insulin resistance^31^. Interestingly, betatrophin has recently been linked to hepatic coexpression of insulin-like growth factor-binding protein 1 (IGFBP-1), a protein prolonging the halftime of IGF-1 and IGF-2 and thereby involved in improving glucose regulation and insulin sensitivity. A slightly weaker coexpression of peroxisome proliferator-activated receptor γ coactivator 1-α (PPARG-C1A), a coactivator of PPARγ has also been shown^32^. Combining the input of these two papers, a connection to the physiologic hyperglycemic state during pregnancy and the coexpression of betatrophin with IGFBP-1 and PPARG-1CA seems plausible. This can also be seen in the present study as the betatrophin concentrations drop postpartum, especially in the obese- and normal weight cohorts. The question remains whether the increased betatrophin levels are, in part, responsible for the body’s response to counteract the reduced insulin sensitivity. A recent study supports this theory by revealing a clear increase in betatrophin expression in response to high insulin levels in cell culture, in a mouse model as well as in T2DM patients receiving insulin treatment^33^ which can be linked to the Akt pathway^33,34^. Interestingly, this theory has also been confirmed by Wang et al., who showed a clear decline in betatrophin concentrations after metformin treatment^24^. As the RYGB cohort in the present study shows the lowest betatrophin levels despite high postprandial insulin levels, other factors need to be taken into consideration. Liu et al.^34^ did not investigate the influence of rapid and pronounced changes in insulin levels, as this is the case in gastric bypass-operated patients in the present study. Also, as betatrophin is an adipose tissue-derived protein, the decrease in visceral adipose tissue after an RYGB operation might also partially explain the lower betatrophin levels in the RYGB cohort. Consistent with this theory, for example, we have recently shown that pregnant RYGB patients have lower levels of liver fat when compared to obese controls^14^.

Our study has limitations which have to be reported. First, the number of participating patients in the three groups was low. However, this was a prospective clinical study and the number of participants is comparable to earlier studies. Another limitation is that especially after pregnancy the drop-out rate was high and therefore the changes in betatrophin levels post-partum are limited to a restricted number of patients. Additionally, the conclusions of the present study are based on correlations and not on causative relationships.

To the best of our knowledge, this is the first study to investigate the changes in betatrophin in pregnant RYGB-operated women; there is no reference to similar results to date. Further research ought to be conducted on this subject in order to precisely make assessments and predictions for the possible implications of these findings. Nevertheless, our results show that betatrophin is downregulated in pregnant women with a history of RYGB operation and that there is a negative association with the disposition index. It is possible that downregulation of basal and dynamic betatrophin levels in RYGB-operated pregnant women is a consequence of the exaggerated risk of postprandial hypoglycaemic events.
